# 3D Printed Multi-Functional Scaffolds Based on Poly(ε-Caprolactone) and Hydroxyapatite Composites

**DOI:** 10.3390/nano11092456

**Published:** 2021-09-21

**Authors:** Fan Liu, Honglei Kang, Zhiwei Liu, Siyang Jin, Guoping Yan, Yunlong Sun, Feng Li, Haifei Zhan, Yuantong Gu

**Affiliations:** 1 School of Materials Science and Engineering, Wuhan Institute of Technology, Wuhan 430205, China; fan.liu@wit.edu.cn (F.L.); polymers20121016@sohu.com (Z.L.); jinsiyang163@163.com (S.J.); 2 Department of Orthopaedics, Tongji Medical College, Huazhong University of Science and Technology, Wuhan 430022, China; kanghonglei@hust.edu.cn (H.K.); dongyimin@hust.edu.cn (Y.S.); 3 Department of Civil Engineering, Zhejiang University, Hangzhou 310058, China; zhan_haifei@zju.edu.cn; 4 School of Mechanical, Medical and Process Engineering, Queensland University of Technology, Brisbane, QLD 4001, Australia; yuantong.gu@qut.edu.au

**Keywords:** poly(ε-caprolactone), hydroxyapatite, biodegradability, 3D printed scaffolds, bone tissue regeneration

## Abstract

3D Printed biodegradable polymeric scaffolds are critical to repair a bone defect, which can provide the individual porous and network microenvironments for cell attachment and bone tissue regeneration. Biodegradable PCL/HA composites were prepared with the blending of poly(ε-caprolactone) (PCL) and hydroxyapatite nanoparticles (HA). Subsequently, the PCL/HA scaffolds were produced by the melting deposition-forming method using PCL/HA composites as the raw materials in this work. Through a serial of in vitro assessments, it was found that the PCL/HA composites possessed good biodegradability, low cell cytotoxicity, and good biocompatibility, which can improve the cell proliferation of osteoblast cells MC3T3-E1. Meanwhile, in vivo experiments were carried out for the rats with skull defects and rabbits with bone defects. It was observed that the PCL/HA scaffolds allowed the adhesion and penetration of bone cells, which enabled the growth of bone cells and bone tissue regeneration. With a composite design to load an anticancer drug (doxorubicin, DOX) and achieve sustained drug release performance, the multifunctional 3D printed PCL/HA/DOX scaffolds can enhance bone repair and be expected to inhibit probably the tumor cells after malignant bone tumor resection. Therefore, this work signifies that PCL/HA composites can be used as the potential biodegradable scaffolds for bone repairing.

## 1. Introduction

Due to the loss of osteogenic microenvironment, it is a great clinical challenge to repair a large bone defect [1,2,3]. The 3D Printed biodegradable polymeric scaffolds possess the individual porous and network structures, which provide the matrix and microenvironment for cell attachment and bone tissue regeneration. In particular, these polymeric scaffolds do not need to be removed by a second operation since polymers can be completely degraded in vivo [4,5,6]. Biodegradable aliphatic polyesters, such as poly(lactic acid) (PLA) and poly(ε-caprolactone) (PCL), are the attractive polymers that have been widely used as the tissue engineering matrix and drug-controlled release system [7,8,9]. Specifically, PCL is a preferred synthetic aliphatic polyester biomaterial due to its good biodegradability, good biocompatibility, low toxicity, and weak inflammatory effect of the degraded products. It has been shown to be suitable for the fabrication of biodegradable scaffolds for tissue regeneration and tissue engineering [10,11,12].

The polymer composites containing nanoceramics were reported to possess better mechanical properties than those of the pure polymers due to additional strength and stiffness provided by the embedded ceramic nanoparticles. As such, the polymer composite-based scaffolds can withstand a certain level of physiological loading and function before the new tissue replaces the scaffold matrix (during its gradual degrading process) [13,14]. Hydroxyapatite (HA) is a naturally occurring mineral form of calcium apatite and an important component of human skeleton and teeth. It has been widely applied as the good bone grafting material and replacement and supplement material because of its convenient production, low toxicity, good biocompatibility, and high bioactivity [15,16].

In the prosthodontic treatment of bone defects, the ideally implanted scaffolds should be biodegradable while new bone tissue is growing. They should have good bioactivity to promote the bone-binding ability, and possess other biological actions such as antitumor, anti-collagenolytic, and anti-inflammatory properties for bone regeneration. Recently, some physical and chemical methods (such as surface functionalization and blending) were used to fabricate functional scaffolds for tissue engineering applications. For instance, Sarkar et al. [17] fabricated calcium phosphate (CaP) scaffolds with the encapsulation of the hydrophobic biomolecule curcumin (within a liposome), which eradicated the osteosarcoma cells and also promoted osteoblast proliferation, offering new opportunities to treat bone defects after tumor resection.

Poly(L-lactic acid) (PLA)/hydroxyapatite (HA) composites were early used as the substitute materials for metal bone plates for bone fixation and for healing bone fractures, which is compatible with the human body and avoids the need for a second operation [18]. However, PLA appears to have the hard, crisp character, poor toughness, bulk degradability, and acid degradation products, which induce local pH reduction and some adverse inflammatory reaction [19]. Thus, it is of great interest to explore whether HA can be applied as reinforcements for PCL in biomedical applications. The modification of PCL by HA induced the inhibition of self-acceleration of acidolysis and decrease of hydrolysis of ester bonds since the alkalinity of HA nanoparticles can neutralize acidic substances during the degradation of ester bonds [20].

This work prepared the polymer composites based on PCL and HA with the addition of doxorubicin (DOX) as an anticancer drug. DOX was chosen as a model drug due to its widespread use in bone cancer. Biodegradable scaffolds were further fabricated based on the polymer composites, using the melting deposition-forming approach. The polymer composites were also evaluated to be used as the potential biodegradable scaffolds for bone repairing. With a composite design to load DOX and achieve sustained drug release, the scaffolds can be expected to enhance bone repair, inhibit probably the tumor cells, and improve patient outcomes.

## 2. Materials and Methods

### 2.1. Materials

All chemicals and solvents were of analytical grade. Toluene and tetrahydrofuran (THF) were purified by redistillation over sodium. Triethylamine was refluxed under phthalic anhydride and dried over calcium hydride (CaH_2_) before use. Tin (II) 2-ethylhexanoate (Sn(Oct)_2_) was purchased from Sigma-Aldrich (St. Louis, MO, USA) and purified by redistillation in vacuo before use. PCL was synthesized by ring-opening bulk polymerization of ε-caprolactone using Sn(Oct)_2_ as a catalyst [21,22,23]. PCL was characterized by the gel permeation chromatography, ^1^H NMR, Fourier transform infrared spectroscopy, UV, differential scanning calorimetry, and automatic contact-angle measurements. PCL: ^1^H NMR (300 MHz, CDCl_3_, δ, ppm): 4.25 (m, 4H, C-CH_2_), 2.06 (m, 2H, C-CH_2_-C). FT-IR (KBr, cm^−1^): 3450 (OH), 2963, 2922 (C-C), 1740 (C=O), 1461 (C-C), 1172, and 1034 (C-O). The molecular weight (Mn) was 1.94 × 10^5^ and polydispersity was 1.47, as determined by Gel Permeation Chromatography (GPC, Waters Corporation Milford, Milford, MA, USA). Hydroxyapatite (HA) nanoparticles with average diameter of particle sizes of 20 nm were purchased from the Beijing Deke Daojin Science and Technology Co., Ltd. (Beijing, China).

### 2.2. Instrumentation

The compounds were characterized using a UV-Vis spectrophotometer (UV-2800 series, Unico, Shanghai, China), a Nicolet is10 Fourier Transform-Infrared (FT-IR) spectrophotometer (Thermo Fisher Scientific Inc., Madison, WI, USA), a Varian Mercury-VX300 NMR spectrometer (Varian, Inc. Corporate, Palo Alto, CA, USA), and an automatic contact angle meter (SL200A/B/D Series, Solon Tech. Inc. Ltd., Shanghai, China). The molecular weight was measured by gel permeation chromatography (Waters 2965D separations module, Waters 2414 Refractive Index Detector, Shodex K802.5 & K805 with Shodex K-G Guard Column, Polystyrene Standard, DMF solvent, 1.0-mL∙min^−1^ flow rate, 323-K Column temperature, and 323-K Detector temperature). The glass transition temperature (Tg) was measured by a differential scanning calorimeter (DSC) (NETZSCH DSC 200 F3, Erich NETZSCH GmbH & Co. Holding KG, Gebrüder-Netzsch-Strasse, Selb, Germany). The morphologies were characterized using a SEM (JEOL JSM-7001F, Akishima City, Tokyo, Japan) at 5–10 kV. Before SEM observation, the specimens were gold sputter-coated using an auto fine coater under argon atmosphere for 60 s. The elemental distribution of calcium (Ca), phosphorus (P), oxygen (O), and carbon (C) in the scaffolds was investigated using an EDS (Phenom World BV, Eindhoven, Netherlands). The phase composition was investigated using a XRD diffractometer (German Bruker Co., Karlsruhe, Germany) with Cu Ka radiation (λ = 0.154056 nm, 40 kV, 40 mA). Before analysis, the scaffold specimens were fixed on a specimen holder by double-side adhesive tape. The data were recorded in the 2θ range of 10–75° with scanning speed of 8° min^−1^. The absorbance (optical density: OD_492_) was measured with a DG-3022A ELISA-Reader (Hercules, CA, USA). The osteoblast cells MC3T3-E1 were provided by the China Center for Type Culture Collection of Wuhan University, China. The ethical approval was obtained for the in vivo experiments in animals from the Department of Science and Technology of Hubei Province, China, and the Animal Center of Tongji Medical College, Huazhong University of Science and Technology, China.

### 2.3. Preparation of Poly(ε-Caprolactone) and Hydroxyapatite (PCL/HA) Scaffolds

Two different types of composite (PCL/HA and PCL/HA/DOX) samples were prepared, with one containing PCL and HA and the other one containing PCL, HA, and doxorubicin (DOX). Dichloromethane was used to ensure an even mixture between PCL and HA (or DOX). The mixtures were slowly evaporated to dry under reduced pressure. The residues were cut into small pieces and dried under vacuum for 48 h to yield PCL/HA composites and PCL/HA/DOX composites.

Thereafter, the cylinder or circular 3D printed PCL/HA scaffolds (diameter: 4 mm and height: 6 mm; diameter: 10 mm and height: 2 mm) and multi-functional PCL/HA/DOX scaffolds containing DOX (diameter: 4 mm and height: 6 mm) were prepared by the melting deposition-forming method using a 3D printer of melting deposition forming (Hubei Joye 3D High-tech Co., Ltd., Chibi, Hubei, China) with PCL/HA and PCL/HA/DOX composites as raw materials, respectively. The printing conditions were listed as follows: The printing temperature was 130 °C and preheating temperature of the operation desk was 50 °C, the nozzle aperture was ≥0.4 mm, the extrusion speed was 220 mm/min, and the printing rate was 70 mm/s. In vivo implanted PCL/HA scaffolds in a thigh bone defect model of lower limb in rabbits were further prepared by the loading of 3D printed PCL/HA scaffolds and multi-functional PCL/HA/DOX scaffolds with collagen I, respectively, according to the methods cited in the literatures [24,25,26,27].

### 2.4. In Vitro Degradation Test

PCL and PCL/HA scaffolds (0.1 g, diameter: 10 mm and height: 2 mm) were prepared as above and then dried in vacuum for 24 h. The scaffolds were suspended in 10 mL of PBS in a dialysis bag. The dialysis bag was sealed and then slowly shaken in 90 mL of PBS (pH 7.4) at 37 °C in a 250 mL Erlenmeyer flask. At predetermined time intervals, the samples were taken out of the degradation medium, rinsed with distilled water, and then dried in vacuo for 48 h. The molecular weight, water absorption, and weight loss were calculated, respectively.

The PCL/HA samples for mechanical testing were further prepared by the thermoforming of PCL/HA composite materials in a standard mold on an injection molding machine. An in vitro degradation test was also measured using PCL/HA samples, according to the above same methods as the PCL/HA scaffolds.

### 2.5. In Vitro Drug Release Study

Doxorubicin (DOX, 10 mg) and PCL or PCL/HA composites (100 mg) were dissolved in 20 mL of THF. The solution was homogenized by sonication for 30 s and then allowed to evaporate. The resulting films were cut into small pieces and dried under vacuum for 48 h to obtain 5-DOX-incorporated PCL or PCL/HA composites. The 5-DOX-incorporated PCL or PCL/HA scaffolds were further prepared by the thermoforming of 5-DOX-incorporated PCL or PCL/HA composites in a disc mold (diameter: 10 mm and height: 2 mm) on a thermocompressor with loading pressure of 10 MPa, setting temperature of 110 °C, and molding time of 10 min.

The 5-DOX-incorporated PCL or PCL/HA scaffolds were suspended in 10 mL of PBS in a dialysis bag. The dialysis bag was sealed and then slowly shaken in 90 mL of PBS at 37 °C in a 250-mL Erlenmeyer flask. Aliquots of the solution outside the dialysis membrane (25 mL) were replaced with 25 mL of PBS at various time intervals and tested at 256 nm by a high-performance liquid chromatography spectrophotometer (HPLC). The changes of the concentrations of DOX were obtained from curves of the absorption A *versus* concentration C of DOX in PBS on the basis of the Lambert-Beer law.

### 2.6. Cell Viability and Proliferation Assay

The circular 3D printed PCL and PCL/HA scaffolds (diameter: 10 mm, thickness: 2 mm, the content of HA: 5%, 10%, 15%, 20%, and 25%) were placed into the wells of 24-well plate. The scaffolds were sterilized using Ultraviolet light for 1 h for each side and secured with a stainless-steel ring. MC3T3-E1 cells were seeded onto the scaffolds at a density of 4 × 10^4^ cells/mL and 100 µL of the RPMI-1640 growth medium was added. Cell counting kit 8 (CCK-8) assay was performed on 1 and 3 days after culturing. The cells were incubated for 1 and 3 days in an incubator (37 °C, 5% CO_2_) and the medium was replaced by the fresh growth medium. Then, 10 µL of CCK-8 solution was added to each well and continued incubating for 3 h. The absorbance (optical density: OD_492_) was measured at 492 nm with a DG-3022A ELISA-Reader and expressed as a percentage relative to control cells (no scaffolds).

### 2.7. In Vivo Implanted Assay of PCL/HA Scaffolds in Skull Defect and Histological Analysis

Eighteen 6-week-old SD rats were divided into three groups (control, PCL, and PCL/HA, for each group, *n* = 6) and the circular skull defect model (diameter of about 10 mm) was made in the head of each rat. Subsequently, the rat was anesthetized with urethane (10%, 10 mL/kg), positioned prone, and fixed to a polystyrene cradle with adhesive tape to minimize motion. The scalp along the sagittal suture was shaved and the skin was sterilized with iodine. A 15-mm-long incision along the sagittal suture was made. Then, the calvaria bone was exposed by blunt dissection. A 10-mm-diameter defect was created using a trephine bur on left parietal bone. The circular scaffold of 10 mm diameter was sterilized with UV light for 1 h for each side and implanted into the bone defects for each group (PCL and PCL/HA scaffolds). For the control group, the rats were treated the same, but without implantation. The periosteum and the overlying skin were then stitched up using the nylon suture. After 1 month postsurgery, the rats were anesthetized and perfused through the heart by using 4% paraformaldehyde. The whole calvaria was harvested and further fixed with 4% paraformaldehyde at room temperature for 2 days for the following evaluations. To assess the new bone formation in the bone defect area, micro-CT was applied, and the area of new bone formation and percentage were measured using the Image Processing and Analysis in Image J-1.52J (WayneRasband National Institutes of Health, Bethesda, MD, USA) software.

Nine 6-week-old SD rats were divided into three groups (control, PCL, and PCL/HA scaffolds, for each group, *n* = 3) and anesthetized with urethane (10%, 10 mL/kg), positioned prone, and fixed to a polystyrene cradle with adhesive tape to minimize motion. The scalp along the sagittal suture was shaved and the skin was sterilized with iodine. A 15-mm-long incision along the sagittal suture was made in the muscle on the back. The circular scaffolds of 10 mm diameter were sterilized with UV light for 1 h for each side and implanted into the muscle defects for each rat (PCL and PCL/HA scaffolds). For the control group, the rats were treated the same, but without implantation. The periosteum and the overlying skin were then stitched up using the nylon suture. After 3 days postsurgery, the rats were anesthetized, and the circular scaffolds were moved. The samples of muscle tissues around the scaffolds were taken out and embedded in paraffin and sectioned at a thickness of 5 mm for Hematoxylin-eosin (H&E) staining. The H&E staining slides were observed under an optical microscope, and the images were captured under an IX-70 fluorescence inverted microscope (Olympus Co., Ltd., Tokyo, Japan). Histological evaluation was performed by two independent examiners.

### 2.8. In Vivo Implanted Assay of PCL/HA Scaffolds in Thigh Bone Defect

The cylinder 3D PCL/HA scaffolds containing collagen I and multi-functional PCL/HA/DOX scaffolds containing collagen I and DOX (diameter: 4 mm and height: 6 mm) were further produced by the loading of 3D printed PCL/HA scaffolds and multi-functional PCL/HA/DOX scaffolds with collagen I, according to the methods in the cited literature, respectively. Eighteen 6-week-old New Zealand white rabbits were divided into three groups (control, PCL/HA scaffolds with 25% HA content loading of collagen I, and multi-functional PCL/HA scaffolds with 25% HA content loading of collagen I and DOX, for each group, *n* = 6) and the circular thigh bone defect model (diameter of about 4 mm) was made in each rabbit. Subsequently, the rabbits were anesthetized with urethane (10%, 10 mL/kg), positioned prone, and fixed to a polystyrene cradle with adhesive tape to minimize motion. The scalp along the sagittal suture was shaved and the skin was sterilized with iodine. A 15-mm-long incision along the sagittal suture was made. Then, the thigh bone was exposed and a 4-mm-diameter defect was created using a trephine bur in the cancellous bone. The scaffolds (diameter: 4 mm and height: 6 mm) were sterilized with UV light for 1 h for each side and implanted into the bone defects for each group. For the control group, the rabbits were treated the same, but without implantation. The periosteum and the overlying skin were then stitched up using the nylon suture. After 2 months postsurgery, the rabbits were anesthetized and perfused through the heart by using 4% paraformaldehyde. The whole thigh bone was harvested and further fixed with 4% paraformaldehyde at room temperature for 2 days for the following evaluations.

The new bone formation in the bone defect area was assessed using micro-computed tomography (micro-CT) (Skyscan1276 X-Ray Microtomograph (Micro CT), Bruker, Karlsruhe, Germany) under the fixed conditions (voltage: 93 Kv, current: 800 μA, scanning resolution: 6.5 μm). The area of new bone formation and percentage were measured using the subsidiary image processing and analysis software in the Skyscan1174 Micro CT Scanner, taking the femoral implant as the reference baseline. The cylindrical area with a diameter of 4.1 mm and a thickness of 6 mm was set as the three-dimensional reconstruction area of interest (ROI). The three-dimensional image was reconstructed and three-dimensional analysis was performed. Moreover, the bone mineral density (BMD) of the new bone area was also measured.

### 2.9. Statistical Analysis

All results were expressed as mean differences and were tested for significance by a *t* test, *p* < 0.05 being considered a significant difference.

## 3. Results and Discussion

### 3.1. Characterization

**Figure 1:**
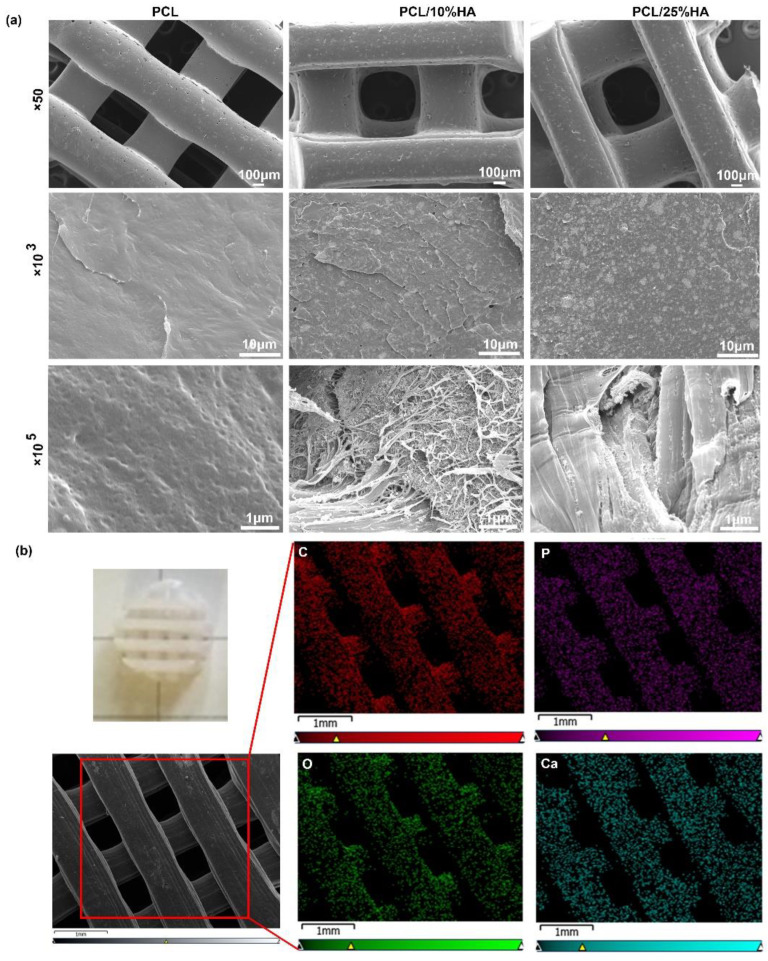
The characteristics of scaffolds. (**a**) Micromorphology of PCL/HA scaffolds in different content (0%, 10%, 25%) (upper panel: ×50, middle panel: ×10^3^, lower panel: ×10^5^); (**b**) The optical graphs and EDS mapping distribution of C, O, Ca, and P elements in the surface of PCL/25% HA scaffolds.

The micrographs of PCL/HA scaffolds were examined by SEM and shown, as in Figure 1a. HA nanoparticles scattered uniformly in the scaffolds with some aggregated occasions. Figure 1b shows the mapping distribution of C, O, Ca, and P elements in the HA powders, which appear uniformly distributed in the scaffolds. Herein, the elemental distribution in the scaffolds was investigated using an EDS. In this work, PCL/HA scaffolds with 400–800 nm of uniform pore sizes were chosen as the implanted samples since such pore size is expected to be beneficial for cell proliferation/differentiation in vivo.

**Figure 2:**
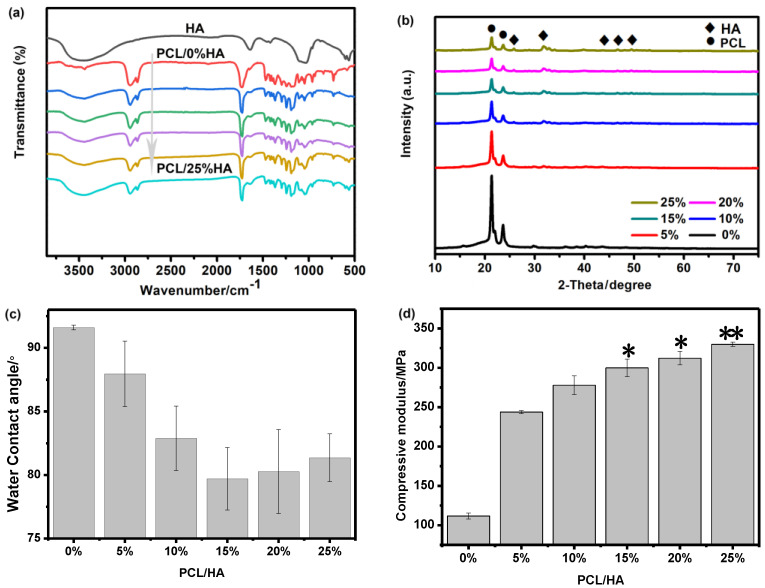
Characterization of different PCL/HA scaffolds with different content of HA, ranging from 0% to 25%. (**a**) FT-IR result; (**b**) XRD spectra; (**c**) Water contact angle; and (**d**) Compressive modulus,* *p* < 0.05, ** *p* < 0.01, vs. PCL.

Chemical characterization of pure PCL, HA, and PCL/HA scaffolds with different content of HA was conducted by FT-IR, and their characteristic peaks are shown in Figure 2a. For the composite samples, many of the absorption bands were overlapped but the properties of the functional groups remained unchanged, whereas the shape, location, and intensity of the spectral peaks changed significantly. The spectra of PCL/HA scaffolds showed typical ester peaks (at 1724 cm^−1^, 1640 cm^−1^) and CH peaks (2923 cm^−1^). The increase of HA content in the scaffolds (from 0 to 25%) decreased the peak intensities. Specifically, PCL/HA scaffolds showed spectral features in the range of 1150 and 960 cm^−1^, which were related to the P-O and P=O vibrations of HA. The peak intensities of the spectral range of 3500-3200 cm^−1^ that represented the absorption peak of OH groups of HA decreased evidently.

**Figure 3:**
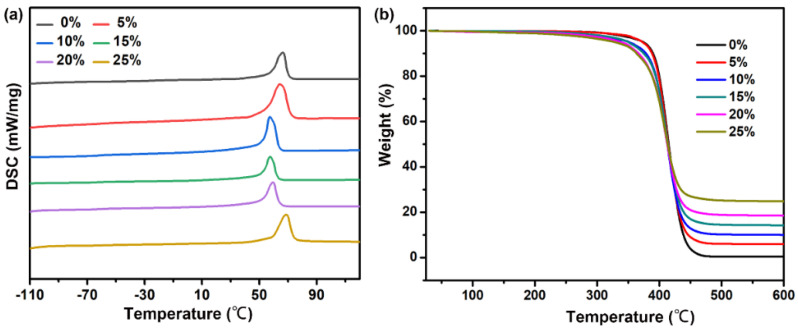
The thermal properties of the PCL/HA scaffolds with different HA contents. (**a**) DSC profiles for the PCL/HA scaffolds with different content of HA; (**b**) Thermogravimetric-differential thermal analysis curves of PCL/HA scaffolds.

Typical X−ray diffraction (XRD) spectra obtain for PCL/HA scaffolds are presented in Figure 2b. The data were recorded in the 2θ range of 10°-75° with a scanning speed of 8 min^−1^. As illustrated in Figure 3b, the spectra of PCL/HA scaffolds appeared similar to those of pure PCL scaffolds. In addition to the strong peaks associated with the crystalline PCL phase, relatively weak peaks at ~25.32°, 32.78°, 41.43°, 45.58°, and 46.58° were observed, which correlated to the crystalline HA phase. These observations implied the presence of both PCL and HA in the mixture composites. Combined with the above SEM image in Figure 2a, these observations indicated that the fine HA nanocrystals were well dispersed in the PCL matrix.

Figure 2c compared the water contact angle of the PCL/HA scaffolds containing different content of HA. The water angle was measured using an automatic contact angle meter. As can be seen, the water contact angle decreased from 91.6° for pure PCL scaffolds to 80.70° for PCL/HA scaffolds with 15% HA content, and then increased gradually to 81.4° when the HA content increased further to 25%. In all examined samples, the water contact angle on the PCL/HA scaffolds was much smaller than that on the pure PCL scaffolds. Such results demonstrated that the presence of nano HA enhanced the surface hydrophilicity of the PCL/HA scaffolds [24,25].

The compressive moduli of PCL/HA scaffolds were measured, as shown in Figure 2d. Here, the scaffolds were fabricated in the form of cylinders (with a diameter of 4 mm and a height of 6 mm) and vertically placed between two parallel plates in a universal testing machine (MTS Industrial Systems Co., Ltd., Shenzhen, China). A compression rate of 1.0 mm/min was utilized. Each compressive test was repeated five times. As is seen, the compressive modulus increased from 112 MPa to 330 MPa when the HA content increased from 0 to 25%. In the literature, the compressive modulus for pure PCL was measured between 85 MPa and 224.9 MPa [26,27], which agreed with our measurements. The enhancement was expected, as originating from the higher intrinsic mechanical properties of HA (an average compressive strength of 174 MPa and a Young’s modulus of 6 GPa) [28] and the uniform distribution of HA in the PCL matrix, which, as shown in Figure 1, play a very important role in the mechanical improvement of PCL/HA scaffolds [29].

The glass transition temperature (Tg) of the PCL/HA scaffolds was measured by DSC. For pure PCL scaffolds, the glass transition temperature (Tg) was −66.4 °C [30]. Figure 3a shows the DSC curves of PCL and PCL/HA composites, for which the Tg varied from −70 °C to −60 °C, which suggested a good compatibility between PCL and HA. According to Figure 3b and Table S1 (see supporting information Table S1), the degradation temperature for weight loss at 10% decreased from 389.7 °C to 363.3 °C when HA content increased from 0% to 25%. Similar results were also observed for the degradation temperature for weight loss at 50% (from 515.0 °C to 414.0 °C). Therefore, the results from thermogravimetric analysis (TGA) indicated that the addition of HA in PCL decreased the thermal stability of scaffolds, as the starting decomposition temperature decreased.

**Figure 4:**
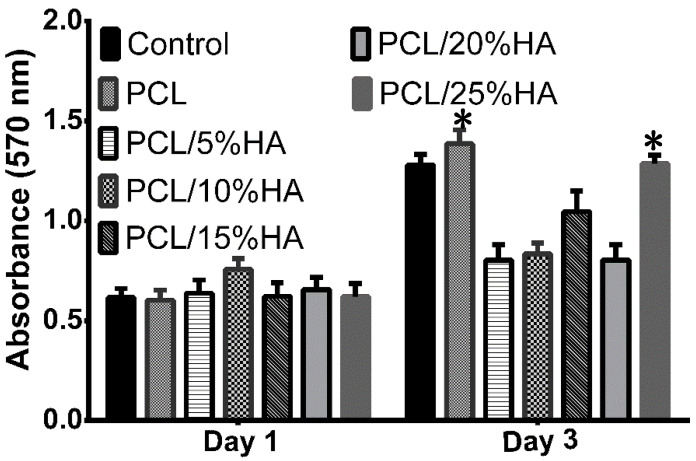
Cell proliferation results of MC3T3-E1 cells after culturing for 1 and 3 days on the PCL/HA scaffolds with 0%−25% HA content. * *p* < 0.05 vs. Control.

Additionally, the in vitro biocompatibility of the PCL/HA scaffolds was carried out in terms of the proliferation of MC3T3-E1 cells. The osteoblast cells MC3T3-E1 were raised, according to the methods described in a literature [31]. In general, no significant differences of cell proliferation activity among all scaffolds were observed after 1 day (Figure 4), whereas the evident differences of cell proliferation activity started to appear among all scaffolds after 3 days of culturing. PCL/HA scaffolds with 25% HA content (PCL/25%HA scaffolds) and pure PCL scaffolds possessed the lower cell cytotoxicity and higher cell proliferation to MC3T3-E1 cells than those of the other scaffolds and the control. Meanwhile, PCL/15% HA scaffolds displayed a higher cell cytotoxicity than those of PCL/5%HA, PCL/10%HA, and PCL/20%HA scaffolds, although PCL/15%HA scaffolds had the lower cell proliferation than those of the control after 3 days of culturing. The cell proliferations on PCL/10%HA scaffolds were slightly higher than those of PCL/5%HA and PCL/20%HA scaffolds. Therefore, these results signified that PCL/25%HA scaffolds possessed good biocompatibility and low cell cytotoxicity and can stimulate cell proliferation well. Consequently, based on the above analysis, PCL/25%HA scaffolds showed the best comprehensive performances and, thus, were selected to assess their biomedical application potentials.

### 3.2. In Vitro Degradation Property of PCL/HA Scaffolds

Ideally, the scaffolds should be biodegradable during the growth of new bone tissue but need to maintain good mechanical properties before the bone tissue is completely regenerated. Thus, it is important to understand the biodegradable behaviors of the scaffolds during the degradation for bone regeneration. For such purpose, PCL and PCL/HA scaffolds were suspended in 10 mL of PBS in a dialysis bag and then slowly shaken in 90 mL of phosphate buffer saline (PBS, pH 7.4) at 37 °C. At predetermined time intervals, the samples were taken out of the degradation medium, rinsed with distilled water, and then dried in vacuo for 48 h.

**Figure 5:**
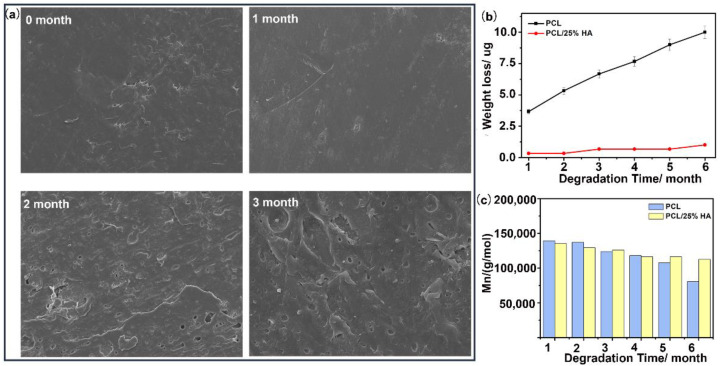
The degradation behaviors of the PCL/25%HA scaffolds in PBS. (**a**) SEM micrographs of PCL/25%HA scaffolds in PBS for 3 months; (**b**) Weight loss and weight-average molecular weight (Mn) of the PCL/HA scaffolds with 0 and 25% HA content after 6 months of degradation.

As illustrated in Figure 5a, the number of large pores and cracks increased in the PCL/HA scaffolds when the degradation time increased, and the PCL/HA scaffolds gradually lost their regularity and uniformity. The degradation of the scaffolds can be well reflected by the weight loss and molecular weight loss shown in Figure 5b,c. Compared with pure PCL scaffolds, the weight losses for PCL/25%HA scaffolds were much smaller during degradation. For instance, after 180 days of degradation, the weight losses of pure PCL and PCL/25%HA scaffolds were about 10% and 1%, respectively. These results indicated that mass loss occurred at a much lower rate for scaffolds with higher HA content. It was probably that the alkalinity of HA nanoparticles induced neutralizing acidic substances during the degradation of PCL, which resulted in the inhibition of self-acceleration of acidolysis and decreasing of hydrolysis of ester bonds.

The Young’s moduli of PCL/25%HA scaffolds after degradation were measured from tensile experiments and summarized in Table S2 and Figure S1 (see Supporting Information Table S2 and Figure S1). It was found that Young’s modulus decreased gradually when the degradation time increased from 0 to 4 months, which aligned with the weight loss during the degradation process. It was expected that the degradation processes mainly occurred at the end and pendant functional groups of the polymeric chain during the first 3 months, and, thus, resulted in a minor influence on the molecular chain. After 90 days, the degradation was expected to induce remarkable reduction to the molecular weight of the polymeric main chain and, thus, result in significant reduction in Young’s modulus.

DSC curves of pure PCL and PCL/25%HA scaffolds after degradation were also measured and are shown in Figure S2 (see Supporting Information Figure S2). Pure PCL and PCL/HA scaffolds were found to keep the characteristic peaks of glass−transition temperature varying from -70 °C to -60 °C after degradation in PBS for 6 months. Specifically, Tg of both samples was found to decrease slightly with the increase of degradation time.

### 3.3. In Vitro Drug-Release Property of PCL/HA Scaffolds

To investigate the drug-release properties of the PCL/HA scaffolds, Doxorubicin (DOX) was chosen as a model drug and mixed in the scaffolds. DOX is a chemotherapy drug that is used to inhibit the growth of tumors cells [29,30,31]. In this work, 10 mg of DOX was mixed with each 100 mg of the pure PCL or PCL/HA composites. After mixture, the samples were evaporated and then shaped in a disc mold (with a diameter of 10 mm and a height of 2 mm) and suspended in 10 mL PBS (pH 7.4) in a dialysis bag with a continuous shaking for 250 h. The DOX loading capacity was determined using UV-Vis spectrophotometer (UV-2800 series, Unico, Shanghai, China) at 483 nm. The changes of the concentrations of DOX were obtained from curves of the absorption *versus* concentration of DOX in PBS following the basis of the Lambert-Beer law [32].

**Figure 6:**
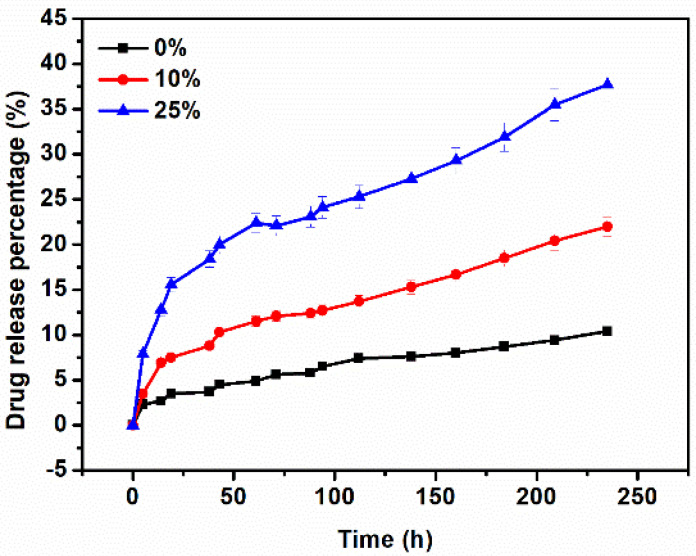
Release profiles of DOX−incorporated PCL/HA scaffolds with 0%, 10%, and 25% of HA content for 250 h.

Recent studies reported that the release amount of DOX at the first 100 h reached over 80% [33,34]. Such a high release rate can inhibit the regeneration of new bone tissue in the tissue engineering. Thus, a controllable release rate is a necessity. Figure 6 compares the DOX-release properties of the pure PCL and PCL/HA scaffolds. After 25 h (or a day), the DOX-incorporated pure PCL and PCL/HA scaffolds displayed steady drug-release rates (as indicated by the linear profile of the curves in Figure 6). Compared with the pure PCL scaffolds, PCL/HA scaffolds exhibited faster drug-release rates, which was suspected as resulting from the increased drug diffusion coefficient (due to the presence of HA). Moreover, the PCL/25%HA scaffolds showed higher release rates than those of the counterpart with 10% of HA. After 34 days, the cumulative DOX-release percentage was around 22.0% and 37.7% for the PCL/HA scaffolds with 10% and 25% HA content, respectively, which were much higher than those of the pure PCL scaffolds (about 10.1%). It was expected that the high content of HA decreased the entanglement degree of PCL, which promoted the DOX release from the scaffolds [35]. The above results suggested that the DOX release rate can be effectively controlled by the content of HA in the PCL/HA scaffolds.

### 3.4. Cell Proliferations of BMSCs in PCL/HA Scaffolds

To further elucidate the effect of PCL/HA scaffolds on the differentiation of rat bone marrow-derived mesenchymal stem cells (BMSCs), the cell proliferation was evaluated using 3-(4, 5-dimethylthiazol-2-yl)-2, 5-diphenyltetrazolium bromide (MTT) assay after culturing for 1 and 2 days, respectively. Cells’ adhesion was performed with 4, 6-diamidino-2-phenylindole (DAPI, Sigma, USA) and FITC-Phalloidin (Sigma, USA), as previously described [36]. The expressions of osteogenic differentiation-related genes and proteins in BMSCs were also investigated. The mRNA transcript levels of Actin, alkaline phosphatase (ALP), collagen (COL), runt-related transcription factor-2 (RUNX2), and osteocalcin (OCN) mRNA within BMSCs (cultured in different supplemented osteogenic−inducing medium) were assessed by real−time polymerase chain reaction (PCR). In both cases, cells were harvested on day 7 and day 14, then lysed in Trizol (Life Technologies, Carlsbad, CA, USA), and mRNA was extracted, according to the manufacturer’s protocol. Reverse transcription was carried out using the RNeasy Plus Micro Kit (Hilden, Germany) and the PCR test was performed using S1000™ Thermal Cycler (Bio−rad Laboratories, Inc., Hercules, USA).

**Figure 7:**
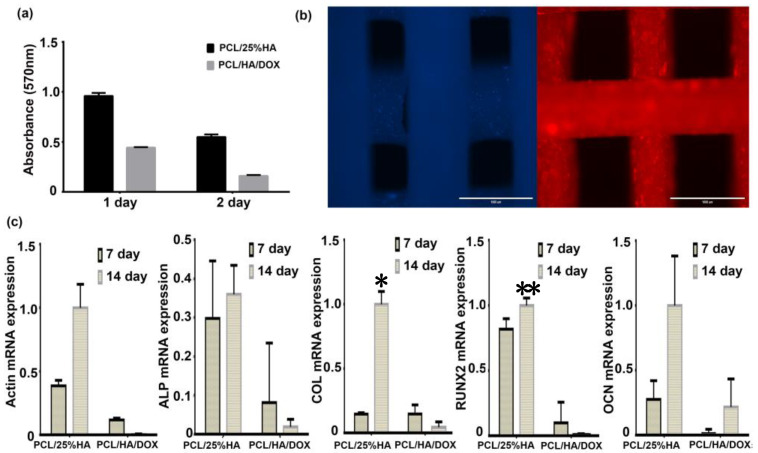
In vitro cell biocompatibility of BMSCs with scaffolds. (**a**) Cell proliferation results of BMSCs after culturing for 1 or 2 days on the PCL/25%HA and PCL/HA/DOX scaffolds, (**b**) Fluorescence morphologies of cells grown on PCL/25%HA scaffolds after 2 days, and (**c**) the expression of Actin, ALP, COL, RUNX2, and OCN mRNA of PCL/25%HA and multi−functional PCL/HA/DOX scaffolds to BMSCs. * *p* < 0.05, ** *p* < 0.01, vs. PCL/25%HA.

As shown in Figure 7a, the cell proliferation activity on PCL/25%HA scaffolds was obviously higher than that of PCL/HA/DOX scaffolds at either day 1 or day 2, whereas the gap between them decreased on day 2. Fluorescence images showed that the BMSCs displayed good adhesion on the surface of the PCL/25%HA scaffolds (Figure 7b), which is beneficial for bone tissue regeneration. From Figure 7c, PCL/25%HA scaffolds supported the growth of BMSCs and evidently promoted the expression of Actin, ALP, COL, RUNX2, OCN mRNA, and proteins. The expressions of those genes and proteins were much higher at day 14 than that at day 7. These results demonstrated that the PCL/25% HA scaffolds possessed good biocompatibility and can stimulate cell proliferation well. Compared with the PCL/25%HA scaffolds, the expressions of those genes and proteins were much smaller in the PCL/HA/DOX scaffolds, which was expected as the reason, resulting from the quickly released DOX.

### 3.5. In Vivo Bone Regeneration with Skull Defect

The in vivo bone regeneration ability of PCL/HA scaffolds was investigated by measuring new bone formation using the model of SD rats with calvaria bone defects. A circular skull defect (with a diameter of 10 mm) was created using a trephine bur (3i Implant Innovation, Palm Beach Gardens, FL, USA) on the left parietal bone of each rat. This size of the defect was chosen because a defect of this size does not heal by itself without intervention. Thereafter, a circular scaffold (with a diameter of 10 mm and a thickness of 2 mm) was sterilized and implanted into the bone defect location for each rat. To assess the new bone formation in the bone defect area after 4 weeks, micro−computed tomography (micro-CT 50, Scanco Medical AG, Bassersdorf, Switzerland) was utilized under the fixed conditions (24 kV, 2 mA, 90 s). Meanwhile, the muscle defects for each rat were made in the muscle on the back. The circular scaffolds of 10 mm diameter were sterilized with UV light for 1 h for each side and implanted into the muscle defects. The muscle tissues around the scaffolds were taken out and embedded in paraffin and sectioned at a thickness of 5 mm for Hematoxylin-eosin (H&E) staining. The H&E staining slides were visualized under an optical microscope.

**Figure 8:**
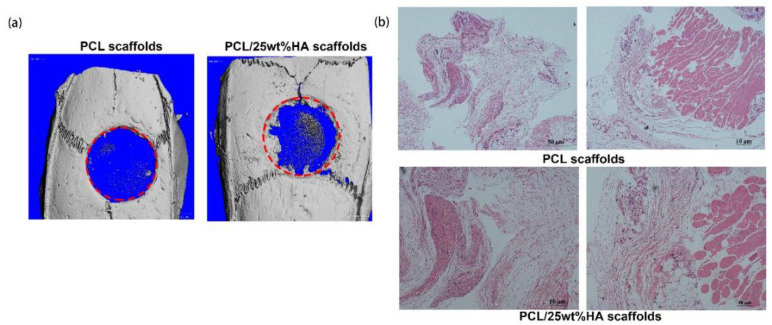
In vivo micro-CT images and histological analysis in skull defect. (**a**) Micro-CT of the skull defect repair in rats with pure PCL and PCL/25%HA scaffolds at 4 weeks; (**b**) H&E staining of the muscle on the back of the rats at 4 weeks post-surgery (HE ×300).

According to the micro−CT data, there was no observable new bone formed in the control group with pure PCL scaffolds after 4 weeks post-surgery (see Figure 8a). In comparison, the group with PCL/25%HA scaffolds showed evident formation of new bone at the edge of the bone defect region (after 4 weeks). In the meantime, there was a certain amount of new bone deposition in the central area of the defects. Further histological analysis affirmed the new bone formation after 4 weeks in the group with PCL/25%HA scaffolds. These observations signified that the PCL/25%HA scaffolds promoted the osteogenic activity, which can repair the bone defect. In the histological analysis, no obvious inflammation was observed in the tissue sections in the H&E staining micrographs after 4 weeks post-surgery (Figure 8b). This observation further suggested that the PCL/25%HA scaffolds possessed good biocompatibility and can provide a good microenvironment for osteoblast proliferation and differentiation.

### 3.6. In Vivo Bone Regeneration with Thigh Bone Defect

The effects of the scaffolds on bone formation in vivo were also evaluated in New Zealand white rabbits with a thigh bone defect in their lower limbs. A circular thigh bone defect (with a diameter of 4 mm) was introduced to the thigh bone of each rabbit. The PCL/25%HA and PCL/HA/DOX scaffolds (with a diameter of 4 mm and a height of 6 mm) were firstly coated with collagen I (see Supporting Information Figure S3) and then sterilized and implanted into the bone defects for each rabbit following the method used in literature [37,38,39,40]. The implanted position was determined by the magnetic resonance imaging using M7 Small animal MRI system (1.0 Tesla, Aspect Imaging Ltd., Israel) (see Supporting Information Figure S4). The area of new bone formation and percentage were measured using the Image Processing and Analysis software, taking the femoral implant as the reference baseline. The cylindrical area with a diameter of 4.1 mm and a thickness of 6 mm was set as the three−dimensional reconstruction area of interest. The three−dimensional image was reconstructed and three−dimensional analysis was performed.

**Figure 9:**
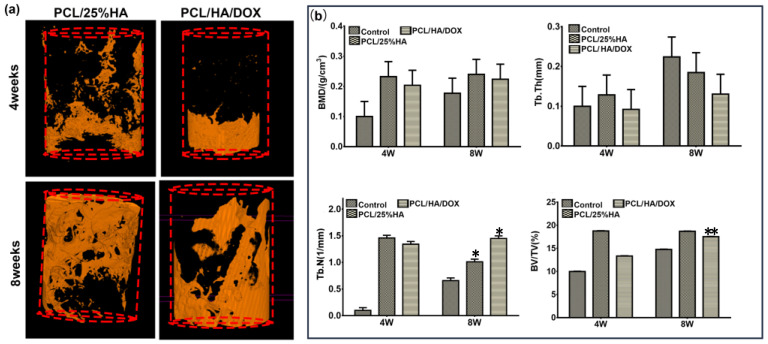
Micro-CT analysis of the effect of scaffolds on thigh bone defect in vivo. (**a**) Representative three−dimensional reconstructed micro-CT images showing the effect of PCL/HA scaffolds on the new bone tissue formation inside the defect site (red, dashed line) (left: PCL/25%HA scaffolds for 4 weeks and 8 weeks, right: PCL/HA/DOX scaffolds for 4 weeks and 8 weeks); (**b**) Summarized data showing the micro-architectural parameters of the newly formed bone tissue at 4 and 8 weeks by analyzing the three-dimensional reconstructed micro-CT images using image analysis software. BMD, BV/TV, Tb.Th and Tb.N are shown in the panel. * *p* < 0.05, ** *p* < 0.01, vs. Control.

As shown in Figure 9, a large amount of new bone formed in the rabbits either with PCL/25%HA scaffolds or PCL/HA/DOX scaffolds after 8 weeks. Bone tissues were found to gradually penetrate into the scaffold, adhere on the scaffold surface, and then grow to form a network. Along with the repair process, the PCL/HA scaffolds degraded gradually. These results indicated both PCL/25%HA and PCL/HA/DOX scaffolds promoted the osteogenic activity and can repair the bone defect. To further assess the newly formed bone, Figure 9b compares bone tissue volume/total tissue volume (BV/TV, %), trabecular thickness (Tb.Th, mm), number of trabecula (Tb.N, mm^−1^), and bone mineral density (BMD, g∙cm^−3^) 8 weeks after surgery. Interestingly, more new bone formation was observed from the rabbits with PCL/25%HA scaffolds than the counterpart with PCL/HA/DOX scaffolds after 4 weeks. Probably the DOX released from the PCL/HA/DOX scaffolds inhibited the cell proliferation activity due to its high cell cytotoxicity to the bone cell during the earlier 4 weeks after implantation. The PCL/25%HA scaffolds were found to result in higher bone volume fraction, larger trabecular thickness, larger number of trabeculae, and higher bone density.

## 4. Conclusions

The biodegradable PCL/HA composites were prepared by the blending of PCL with HA and then multifunctional 3D printed PCL/HA scaffolds were produced by the melting deposition-forming method using PCL/HA composites and a chemotherapy drug (DOX) as raw materials in this work. In vitro assessments revealed that the PCL/HA scaffolds possessed good biodegradability and biocompatibility and promoted cell proliferation. With the addition of DOX, it was found that the PCL/HA/DOX scaffolds had a steady drug release performance, and the release rate can be effectively controlled by the HA content. Subsequently, in vivo experiments for the rats with skull defects and rabbits with thigh bone defects revealed that the PCL/HA and PCL/HA/DOX scaffolds allowed the penetration of bone cells, which enabled the growth of bone cells and bone tissue regeneration. Therefore, the results suggested that PCL/HA composites can be expected as ideal biodegradable scaffolds for bone repairing.

## Data Availability

The data that support the findings of this study are available from the corresponding author upon reasonable request.

## Supplementary Materials

The following are available online at www.mdpi.com/article/10.3390/nano11092456/s1, Table S1: Thermo properties of PCL/HA scaffolds. Table S2: Mechanical properties of PCL/25%HA scaffolds after degradation. Figure S1: Stress-strain curves of PCL/HA scaffolds after degradation. Figure S2: DSC curves of pure PCL and PCL/25%HA after degradation. Figure S3: The characteristics of scaffolds with coating Collagen I. Figure S4: Magnetic resonance imaging of the position of implanted PCL/HA scaffolds in the thigh-bone of rabbit.

## References

[1] Ngo T.D., Kashani A., Imbalzano G., Nguyen K.T., Hui D. (2018). Additive manufacturing (3D printing): A review of materials, methods, applications and challenges. Compos. Part B.

[2] Matai I., Kaur G., Seyedsalehi A., McClinton A., Laurencin C.T. (2020). Progress in 3D bioprinting technology for tissue/organ regenerative engineering. Biomaterials.

[3] Lian H., Zhang L., Meng Z. (2018). Biomimetic hydroxyapatite/gelatin composites for bone tissue regeneration: Fabrication, characterization, and osteogenic differentiation in vitro. Mater. Des..

[4] Yan Y., Chen H., Zhang H., Guo C., Yang K., Chen K., Cheng R., Qian N., Sandler N., Zhang Y.S. (2019). Vascularized 3D printed scaffolds for promoting bone regeneration. Biomaterials.

[5] Bunpetch V., Zhang X., Li T., Lin J., Maswikiti E.P., Wu Y., Cai D., Li J., Zhang S., Wu C. (2019). Silicate-based bioceramic scaffolds for dual-lineage regeneration of osteochondral defect. Biomaterials.

[6] Zhuang P., Sun A.X., An J., Chua C.K., Chew S.Y. (2018). 3D neural tissue models: From spheroids to bioprinting. Biomaterials.

[7] Daly A.C., Pitacco P., Nulty J., Cunniffe G.M., Kelly D.J. (2018). 3D printed microchannel networks to direct vascularisation during endochondral bone repair. Biomaterials.

[8] Jang J., Park J.Y., Gao G., Cho D.W. (2018). Biomaterials-based 3D cell printing for next-generation therapeutics and diagnostics. Biomaterials.

[9] Hu B., Zhai M.Y., Yan G.P., Zhuo R.X., Wu Y., Fan C.L. (2014). Polycarbonate magnetic microspheres containing tumor necrosis factor-α for potential targeted hepatic carcinoma therapeutics. J. Drug Deliv. Sci. Tec..

[10] Hu B., Du H.J., Yan G.P., Zhuo R.X., Wu Y., Fan C.L. (2014). Magnetic polycarbonate microspheres for tumor-targeted delivery of tumor necrosis factor. J. Drug Deliv..

[11] Liu D., Nie W., Li D., Wang W., Zheng L., Zhang J., Zhang J., Peng C., Mo X., He C. (2019). 3D printed PCL/SrHA scaffold for enhanced bone regeneration. Chem. Eng. J..

[12] Wang Q., Yang X., Wang G., Wan L., Wang S., Niu X., Wu J., Pan J. (2020). Osteogenic growth peptide-loaded 3D-printed PCL scaffolds for the promotion of osteogenesis through the ERK pathway. Mater. Design.

[13] Güney A., Malda J., Dhert W.J., Grijpma D.W. (2017). Triblock copolymers based on ε-caprolactone and trimethylene carbonate for the 3D printing of tissue engineering scaffolds. Int. J. Artif. Organs.

[14] Huang K.H., Lin Y.H., Shie M.Y., Lin C.P. (2018). Effects of bone morphogenic protein-2 loaded on the 3D-printed MesoCS scaffolds. J. Formosan. Med. Assoc..

[15] Chen L., Deng C., Li J., Yao Q., Chang J., Wang L., Wu C. (2019). 3D printing of a lithium-calcium-silicate crystal bioscaffold with dual bioactivities for osteochondral interface reconstruction. Biomaterials.

[16] Ma H., Feng C., Chang J., Wu C. (2018). 3D-printed bioceramic scaffolds: From bone tissue engineering to tumor therapy. Acta Biomater..

[17] Sarkar N., Bose S. (2019). Liposome-encapsulated curcumin-loaded 3D printed scaffold for bone tissue engineering. Acs. Appl. Mater. Interfaces.

[18] Leksakul K., Phuendee M. (2018). Development of hydroxyapatite-polylactic acid composite bone fixation plate. Sci. Eng. Compos. Mater..

[19] Zhang B.Q., Wang L., Song P., Pei X., Sun H., Wu L.N., Zhou C.C., Wang K.F., Fan Y.J., Zhang X.D. (2021). 3D printed bone tissue regenerative PLA/HA scaffolds with comprehensive performance optimizations. Mater. Des..

[20] Hutchinson D.J., Granskog V., von Kieseritzky J., Alfort H., Stenlund P., Zhang Y.N., Arner M., Håkansson J., Malkoch M. (2021). Highly Customizable Bone Fracture Fixation through the Marriage of Composites and Screws. Adv. Funct. Mater..

[21] Chen H., Yan G.P., Li L., Ai C.W., Yu X.H.J. (2009). Synthesis, characterization, and properties of ε-caprolactone and carbonate copolymers. Appl. Polym. Sci..

[22] Li D., Zhang K., Shi C., Liu L., Yan G., Liu C., Zhou Y., Hu Y., Sun H., Yang B. (2018). Small molecules modified biomimetic gelatin/hydroxyapatite nanofibers constructing an ideal osteogenic microenvironment with significantly enhanced cranial bone formation. Int. J. Nanomed..

[23] Lu K., Yan G., Chen H., Li L., Ai C., Yu X. (2009). Microwave-assisted ring-opening copolymerization of ɛ-caprolactone and 2-Phenyl-5, 5-bis (oxymethyl) trimethylene carbonate. Chin. Sci. Bull..

[24] Xia Y., Zhou P., Cheng X., Xie Y., Liang C., Li C., Xu S. (2013). Selective laser sintering fabrication of nano-hydroxyapatite/poly-ε-caprolactone scaffolds for bone tissue engineering applications. Int. J. Nanomed..

[25] Medeiros G.S., Muñoz P.A., de Oliveira C.F., da Silva L.C., Malhotra R., Gonçalves M.C., Rosa V., Fechine G.J.J. (2020). Polymer Nanocomposites Based on Poly(ε-caprolactone), Hydroxyapatite and Graphene Oxide. Polym. Environ..

[26] Li Y., Han C., Yu Y., Xiao L. (2020). Effect of loadings of nanocellulose on the significantly improved crystallization and mechanical properties of biodegradable poly(ε-caprolactone). Int. J. Biol. Macromol..

[27] Choi W.Y., Kim H.E., Oh S.Y., Koh Y.H. (2010). Synthesis of poly (ε-caprolactone)/hydroxyapatite nanocomposites using in-situ co-precipitation. Mater. Sci. Eng. C Mater..

[28] Martin R., Brown P. (1995). Mechanical properties of hydroxyapatite formed at physiological temperature. J. Mater. Sci. Mater. Med..

[29] Corcione C.E., Gervaso F., Scalera F., Padmanabhan S.K., Madaghiele M., Montagna F., Sannino A., Licciulli A., Maffezzoli A. (2019). Highly loaded hydroxyapatite microsphere/PLA porous scaffolds obtained by fused deposition modelling. Ceram. Int..

[30] Ramírez Agudelo R., Scheuermann K., Gala García A., Monteiro A.P.F., Pinzón-García A.D., Cortés M.E., Sinisterra R.D. (2018). Hybrid nanofibers based on poly-caprolactone/gelatin/hydroxyapatite nanoparticles-loaded Doxycycline: Effective anti-tumoral and antibacterial activity. Mater. Sci. Eng. C Mater..

[31] Shi F. (1990). Medical Animal Experiment Method..

[32] Javanbakht S., Namazi H. (2018). Doxorubicin loaded carboxymethyl cellulose/graphene quantum dot nanocomposite hydrogel films as a potential anticancer drug delivery system. Mater. Sci. Eng. C Mater..

[33] Kozlu S., Sahin A., Ultav G., Yerlikaya F., Calis S., Capan Y. (2018). Development and in vitro evaluation of doxorubicin and celecoxib co-loaded bone targeted nanoparticles. J. Drug Deliv. Sci. Technol..

[34] Lai Y.L., Cheng Y.M., Yen S.K. (2019). Doxorubicin - chitosan - hydroxyapatite composite coatings on titanium alloy for localized cancer therapy. Mater. Sci. Eng. C Mater..

[35] Ouyang L., Sun Z., Wang D., Qiao Y., Zhu H., Ma X., Liu X. (2018). Smart release of doxorubicin loaded on polyetheretherketone (PEEK) surface with 3D porous structure. Colloid Surf. B.

[36] Yunlong K.H.S., Kesheng L., Hanfeng G., Jie L., Feng L. (2019). Evaluation of mechanical properties and osteogenesis ability of porous titanium alloy scaffolds manufactured by selective laser melting technique. Orthop. Biomech. Mater. Clin. Study.

[37] Du Y.W., Zhang L.N., Hou Z.T., Ye X., Gu H.S., Yan G.P., Shang P. (2014). Physical modification of polyetheretherketone for orthopedic implants. Front. Mater. Sci..

[38] Du Y.W., Zhang L.N., Ye X., Nie H.M., Hou Z.T., Zeng T.H., Yan G.P., Shang P. (2015). In vitro and in vivo evaluation of bone morphogenetic protein-2 (BMP-2) immobilized collagen-coated polyetheretherketone (PEEK). Front. Mater. Sci..

[39] Feng P., Wu P., Gao C., Yang Y., Guo W., Yang W., Shuai C. (2018). A multimaterial scaffold with tunable properties: Toward bone tissue repair. Adv. Sci..

[40] Liu Y., Li T., Ma H., Zhai D., Deng C., Wang J., Zhuo S., Chang J., Wu C. (2018). 3D-printed scaffolds with bioactive elements-induced photothermal effect for bone tumor therapy. Acta Biomater..

