# Supplementary material for: 3D Printed Multi-Functional Scaffolds Based on Poly(ε-Caprolactone) and Hydroxyapatite Composites
Source: Nanomaterials (Basel). 2021 Sep 21;11(9):2456. doi: 10.3390/nano11092456 (PMC8465550; doi:10.3390/nano11092456)
Supplement: Supplementary file 1 [file nanomaterials-11-02456-s001.zip › nanomaterials-1364611-Supplementary Materials (new).pdf]

nanomaterials

Supporting Information

3D Printed Multi-Functional Scaffolds Based on Poly(ε-Caprolactone) and Hydroxyapatite Composites

Fan Liu [1,†], Honglei Kang [2,†], Zhiwei Liu [1], Siyang Jin [1], Guoping Yan [1,*], Yunlong Sun [2], Feng Li [2,*], Haifei Zhan [3,4] and Yuantong Gu [4]

[1] School of Materials Science and Engineering, Wuhan Institute of Technology, Wuhan 430205, China; fan.liu@wit.edu.cn (F.L.); polymers20121016@sohu.com (Z.L.); jinsiyang163@163.com (S.J.)
[2] Department of Orthopaedics, Tongji Medical College, Huazhong University of Science and Technology, Wuhan 430022, China; kanghonglei@hust.edu.cn (H.K.); dongyimin@hust.edu.cn (Y.S.)
[3] Department of Civil Engineering, Zhejiang University, Hangzhou 310058, China; zhan_haifei@zju.edu.cn
[4] School of Mechanical, Medical and Process Engineering, Queensland University of Technology, Brisbane QLD 4001, Australia; yuantong.gu@qut.edu.au
* Correspondence: guopingyan@wit.edu.cn (G.Y.); lifengmd@hust.edu.cn (F.L.) Tel.: +86-27-65-520-576 (G.Y.)
† These authors contributed equally to this work.

S1. The thermo properties of pure PCL and PCL/HA scaffolds

Thermogravimetric analysis (TGA) was carried out to investigate the thermal properties of the scaffolds. Before measurement, the scaffolds were cut into short pieces, and the specimens with a weight of ≈10 mg were placed in an aluminum crucible. The measurements were carried out over a temperature range of 25°C–600°C at a heating rate of 10°C min^{-1}. Table S1 lists the thermal properties of the PCL/HA scaffolds.

Table S1. Thermo properties of PCL/HA scaffolds. Here, $T_{10\%}$, $T_{50\%}$, $T_{80\%}$ and $T_{\approx 100\%}$ represent the degradation temperature at the weight loss of 10%, 50%, 80%, and ≈100%, respectively.

Heating rate (°C/min)	$T_{10\%}$ (°C)	$T_{50\%}$ (°C)	$T_{80\%}$ (°C)	$T_{\approx 100\%}$ (°C)	Residue at 400°C (%)	Residue at 450°C (%)
PCL	389.7	415.0	430.3	492.2	79.53	3.88
PCL/5%HA	388.6	415.2	432.0	501.3	79.02	9.14
PCL/10%HA	378.8	414.1	434.0	500.4	74.28	13.00
PCL/15%HA	375.0	414.6	439.9	529.7	73.90	17.09
PCL/20%HA	367.1	413.7	460.8	533.8	70.03	21.05
PCL/25%HA	363.3	414.0	—	558.0	68.97	27.06

S2. *In vitro* degradation property of PCL/HA scaffolds

The PCL/HA samples for mechanics testing are further prepared by the thermoforming of PCL/HA composite materials in a standard mold (dumbbell shape) on an injection molding machine. *In vitro* degradation test is also measured using PCL/HA samples according to the above same method as PCL/25%HA scaffolds. Tensile properties are evaluated using a universal testing machine at a stretching rate of 1 mm/min. Each experiment is repeated three times and the results are averaged. The tensile properties of PCL/25%HA scaffolds after degradation are measured and summarized in Table S2.

Table S2. Mechanical properties of PCL/25%HA scaffolds after degradation.

Degradation time (days)	Young's modulus (MPa)
30	307.23±2.35
60	290.90±5.65
90	300.00±4.65

120	264.96±3.40

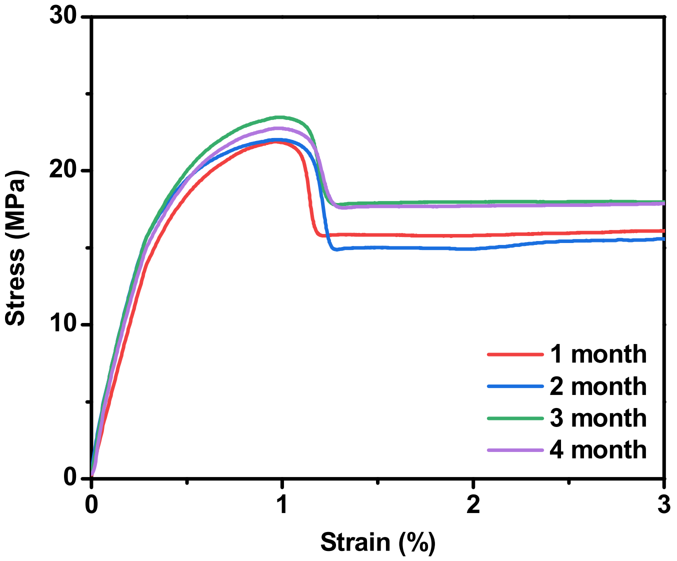

Figure S1. Stress-strain curves of PCL/HA scaffolds after degradation.

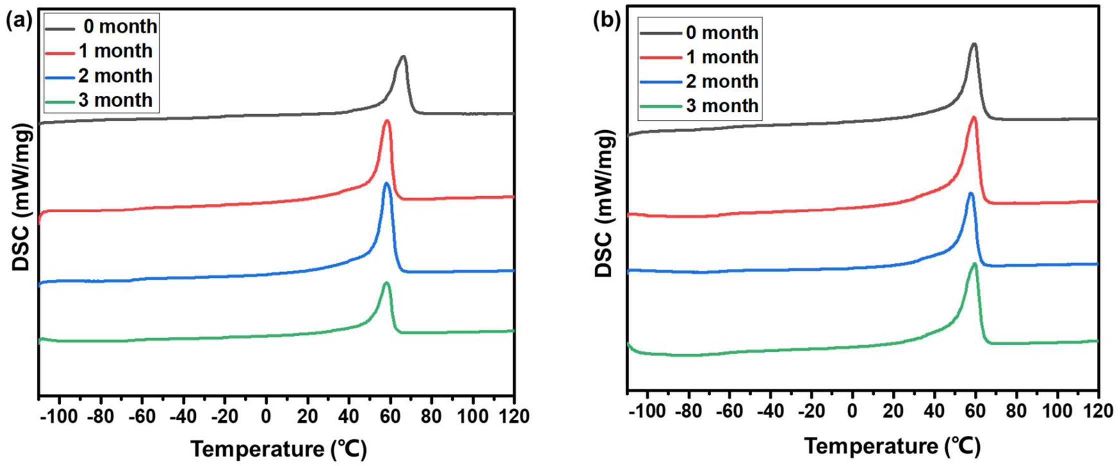

Figure S2. DSC curves of pure PCL and PCL/25%HA after degradation. (**a**) pure PCL scaffolds; and (**b**) PCL/25% HA.

S3. Characterization of PCL/HA/DOX scaffolds coated with collagen I

The SEM images clearly show the surface condition of multi-functional PCL/HA/DOX scaffolds (Figure S3). Limited by the resolution of SEM, the structure of small triple helix can not be seen clearly, but collagen fibrils are observable with the diameter of about 100 nm. The appearance of N_{1s} absorption peak in the survey X-ray photoelectron spectroscopy (XPS) spectrum at 398 eV indicates that the collagen I is successfully coated onto the surface of PCL/HA/DOX scaffolds (Figure S3b). Such observation is further affirmed from the XPS analysis on other three sample discs.

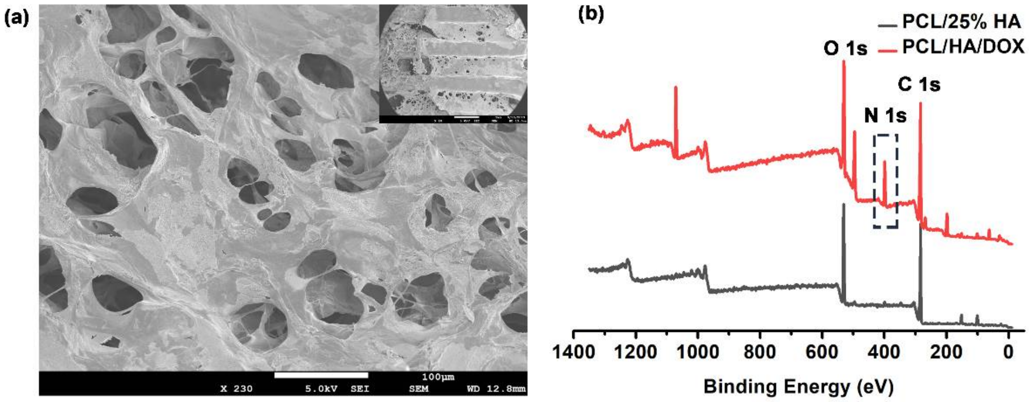

Figure S3. The characteristics of scaffolds with coating Collagen I. (**a**) SEM images of Collagen fibrils were obvious coating on the surface of PCL/HA scaffolds; (**b**) The survey XPS spectra of PCL/25%HA and PCL/HA/DOX scaffolds.

S4. Magnetic resonance imaging of the position of implanted PCL/HA scaffolds in the thigh-bone of rabbit

The MRI images show the multi-functional PCL/HA/DOX scaffolds that were implanted in the thigh-bone of rabbits (Figure S4).

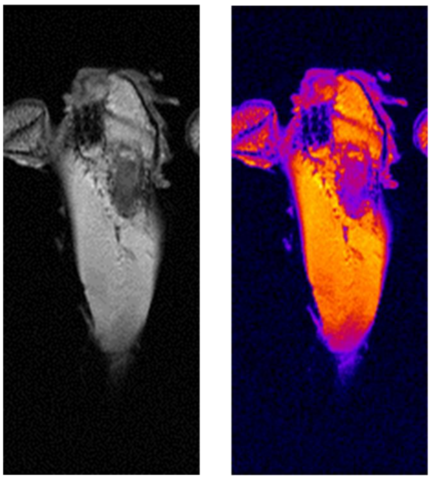

Figure S4. Magnetic resonance imaging of the position of implanted PCL/HA scaffolds in the thigh-bone of rabbits.
